# Nurses’ Perspectives on the Sleep Quality of Hospitalized Patients in Al Ahsa, Saudi Arabia

**DOI:** 10.3390/nursrep15020054

**Published:** 2025-02-04

**Authors:** Rabie Adel El Arab, Husam Alzghoul, Mohammad S. Abu-Mahfouz, Zainab Aldarwish, Mariam Abbadi, Eman Ghashi, Ghasaq Alsaffar, Wujd Alasmkh, Mohamed Mahmoud Seweid

**Affiliations:** 1 Department of Health Management and Informatics, Almoosa College of Health Sciences, Al Ahsa 36422, Saudi Arabia; 2 Department of Nursing, Almoosa College of Health Sciences, Al Ahsa 36422, Saudi Arabia; h.alzghoul@almoosacollege.edu.sa (H.A.); m.abumahfouz@almoosacollege.edu.sa (M.S.A.-M.); 2010120@almoosacollege.edu.sa (Z.A.); 2010069@almoosacollege.edu.sa (M.A.); 2010024@almoosacollege.edu.sa (E.G.); 2010014@almoosacollege.edu.sa (G.A.); 2010126@almoosacollege.edu.sa (W.A.); m.seweid@almoosacollege.edu.sa (M.M.S.); 3 Faculty of Nursing, Beni-Suef University, Beni-Suef 62111, Egypt

**Keywords:** sleep quality, nursing, hospitalized patients, nurses’ perspectives, Roy’s adaptation model, Saudi Arabia, health technology, cultural sensitivity

## Abstract

Background: Sleep quality is crucial for patient recovery and well-being, yet hospitalized patients often suffer from poor sleep due to environmental disruptions, clinical routines, and psychosocial stressors. While these challenges are well-documented, qualitative insights into nurses’ perspectives—essential frontline providers shaping the sleep environment—are scarce, especially within rapidly evolving healthcare systems like Saudi Arabia’s. This study explores nurses’ perceptions of factors influencing patient sleep quality in a private hospital in Al Ahsa, Saudi Arabia, and identifies strategies for improvement. Methods: We conducted a qualitative, cross-sectional study using semi-structured interviews with 14 registered nurses from diverse nationalities, specialties (Obstetrics/Gynecology, Medical-Surgical, Pediatrics, Intensive Care, Orthopedics, Bariatrics), and experience levels. Interviews were conducted in Arabic or English, audio-recorded, transcribed, and thematically analyzed using ATLAS.ti software. Roy’s Adaptation Model guided the examination of environmental, patient-specific, and systemic factors affecting sleep. Findings: Four primary themes emerged: (1). Environmental Factors: noise from alarms, equipment, family presence, and late-night activities, along with abrupt lighting changes, consistently disrupted sleep. (2). Patient-Specific Factors: pain, emotional distress, cultural expectations, and family involvement influenced sleep experiences. (3). Systemic and Contextual Factors: language barriers, infrastructural disparities between private and governmental hospitals, and limited resources can impeded effective sleep-promoting strategies. (4). Role of Health Technology: nurses recognized the potential of innovations like smart lighting and wearable monitors to enhance sleep quality but faced challenges in implementation due to knowledge gaps and limited familiarity. Roy’s Adaptation Model highlighted how effective adaptation through physiological and cognitive–emotional pathways, as observed by nurses, was facilitated or hindered by these factors. Conclusions: Enhancing in-hospital sleep quality requires a holistic, culturally sensitive approach that integrates environmental modifications, patient-centered care, and systemic improvements. Strategic investments in staff communication training, infrastructural upgrades, language support services, and the adoption of health technologies can promote adaptive responses and optimize patient rest. By leveraging theory-driven insights and context-specific strategies, healthcare systems—particularly those undergoing rapid development—can better support nurses in fostering restorative sleep environments as a fundamental component of patient-centered care, thereby enhancing patient recovery, satisfaction, and overall well-being.

## 1. Introduction

Sleep is fundamental to human health, daily functioning, and overall well-being. Its critical role in patient recovery and healthcare has been recognized for centuries, with historical figures like Florence Nightingale emphasizing the importance of restful sleep-in enhancing patient outcomes [1,2]. In the context of hospitalized patients, adequate sleep is paramount for facilitating recovery, enhancing immune function, and reducing the risk of complications [3,4]. However, the hospital environment is often fraught with factors that can disrupt sleep, including noise, light, frequent medical interventions, and the psychological stress associated with illness [5]. These disturbances not only impede patient recovery but also contribute to increased hospital stays, elevated healthcare costs, and diminished patient satisfaction [6].

In Saudi Arabia, the healthcare system has undergone significant transformation over the past decade, characterized by rapid expansion and modernization to meet the growing demands of a diverse and expanding population [7,8]. Despite these advancements, maintaining optimal sleep environments within hospitals remains a challenge. The poor sleep quality in hospital settings can exacerbate medical conditions, delay healing processes, and negatively impact the overall patient experience [6].

Nurses play a critical role in the management and promotion of patient sleep quality. As frontline healthcare providers, nurses are uniquely positioned to implement and advocate for sleep-promoting practices, such as minimizing nighttime disruptions, optimizing medication administration schedules, and creating a conducive sleep environment [9,10,11]. Their insights and perceptions are invaluable in identifying barriers to effective sleep management and developing strategies to enhance sleep hygiene in clinical settings [1]. Despite the recognized importance of sleep quality, there is a paucity of qualitative research exploring the perspectives of Saudi nurses on this issue, particularly within the context of the Kingdom’s evolving healthcare landscape.

The existing literature underscores the multifaceted nature of sleep quality in hospitals, highlighting factors such as environmental noise, lighting, patient–nurse interactions, and staffing levels as significant determinants [12,13]. Cultural considerations also play a vital role, as societal norms and patient expectations may influence perceptions of sleep and rest [14].

Extensive quantitative research has examined sleep quality among hospitalized patients across various countries, addressing multiple dimensions but often neglecting the nuanced, subjective experiences inherent to sleep. Despite sleep being a profoundly personal experience, most studies have employed primarily empirical methodologies, thereby overlooking critical aspects of sleep quality and lacking in-depth exploration and understanding [15,16,17,18,19,20,21]. These approaches often overlook the subjective, context-bound nature of the sleep experience, thereby missing opportunities to tailor interventions that respect cultural sensitivities, language preferences, and resource disparities. To bridge this gap, the present qualitative study seeks to explore the perspectives of nurses regarding the sleep quality of hospitalized patients in Saudi Arabia.

Understanding nurses’ perspectives is crucial not only for enhancing patient care but also for fostering a supportive work environment for nursing staff. Nurses’ capacity to prioritize and implement sleep-promoting strategies can be influenced by various factors.

## 2. Conceptual Framework: Roy’s Adaptation Model

The use of Roy’s Adaptation Model provides a robust framework for understanding how patients and nurses adapt to environmental stimuli that affect sleep quality [22,23]. According to this model, individuals are viewed as biopsychosocial systems that continuously interact with their environment, and their responses to these stimuli are mediated through coping mechanisms that can be categorized as either regulator or cognator subsystems. In the context of this study, the model guides the exploration of how both patients and nurses perceive and adapt to disruptions in sleep quality within the hospital environment.

This model is particularly relevant for analyzing nurse–patient interactions concerning sleep quality. By framing patients and nurses as adaptive systems, the study can systematically investigate how these individuals identify, interpret, and respond to factors that disrupt sleep, such as noise, light, or medical interventions at night. The regulator subsystem, which encompasses physiological responses, can be used to understand how patients’ bodies react to sleep disruptions, while the cognator subsystem, involving cognitive and emotional processes, helps explore how patients and nurses mentally and emotionally cope with these challenges.

Moreover, Roy’s Adaptation Model enables the study to differentiate between adaptive and maladaptive responses, thereby providing a structured approach to identifying strategies that nurses can use to mitigate sleep disturbances and promote better sleep quality for patients [24]. Roy’s Adaptation Model was employed to systematically examine how both physiological and cognitive adaptations occur in response to various stimuli affecting patient sleep quality. By categorizing the factors influencing sleep into environmental, patient-specific, and systemic domains, the model facilitated a structured analysis of how nurses perceive and address these challenges in their daily practice. This integration of the model into the study’s research objectives ensures that the analysis of nurse–patient interactions is both comprehensive and aligned with a well-established theoretical framework.

### 2.1. AIM

The aim of this study is to explore the factors influencing sleep quality among hospitalized patients in Al Ahsa, Saudi Arabia.

### 2.2. Objectives

To identify and describe the key factors affecting sleep quality among hospitalized patients in Al Ahsa, as perceived by nurses.

To explore the strategies currently used by nurses to enhance sleep quality in hospitalized patients.

## 3. Methods

### 3.1. Study Design

This study employed a qualitative, cross-sectional design utilizing semi-structured interviews to explore nurses’ perspectives on factors influencing sleep quality among hospitalized patients. The qualitative approach facilitates exploration of nurses’ lived experiences, offering nuanced insights into their perceptions and practices regarding patient sleep management. This methodology uncovers complex dynamics and personal narratives that quantitative methods may fail to capture, thereby providing a comprehensive understanding of strategies employed by nurses in enhancing patient sleep quality [25,26]. Additionally, the qualitative approach was chosen to illuminate cultural, linguistic, and environmental complexities often obscured by quantitative methods. The research team meticulously developed a semi-structured interview guide in English (Supplementary Materials), informed by the existing literature and collaborative discussions within the team. To ensure linguistic accuracy and cultural relevance, the guide was translated into Arabic and back translated by two bilingual speakers, ensuring that the Arabic version accurately reflected the original English guidelines [27]. The interview guide was meticulously designed to align with the study’s objectives and Roy’s Adaptation Model. Each question was crafted to elicit comprehensive responses related to environmental, patient-specific, and systemic factors affecting sleep quality. To ensure consistency across cross-cultural interviews, all interviewers underwent training sessions focused on cultural competence and standardized interviewing techniques. Additionally, a detailed protocol was established to maintain uniformity in interview administration, regardless of the language used.

### 3.2. Data Collection

A purposive sampling technique was employed to select participants who met specific criteria relevant to the research objectives [28]. Data were collected through semi-structured interviews conducted by the principal investigator (RA). Prior to scheduling interviews, participants received an invitation to the study including the information sheet and a booking link created using Calendly, which was shared through internal hospital email. To maximize participation, two reminders were sent: the first was sent one week before the scheduled interview at the end of October 2024 and the second in mid November 2024. Each interview was conducted face-to-face in designated quiet and comfortable areas within the hospital, ensuring minimal interruptions and providing a supportive environment for open and honest dialogue. Interviews were audio-recorded with the participants’ consent. This setting and approach ensured effective communication and comprehensive data collection. During data collection, the interviewer engaged in reflexive journaling and regularly revisited the interview guide to ensure that language nuances, cultural expectations, and researcher assumptions did not bias the participants’ responses.

### 3.3. Population

The study targeted registered nurses working in inpatient wards caring for patients across all age groups at a private hospital. Inclusion criteria required that nurses have at least one year of experience in inpatient care and regularly work night shifts, defined as being assigned to at least six night shifts per month. This criterion ensured that participants had substantial experience with the unique challenges of managing sleep quality in hospitalized patients during night hours. Nurses who did not meet these requirements or had less than one year of experience were excluded to maintain focus on professionals with significant and relevant experience. This private hospital, characterized by mostly single-patient rooms and advanced infrastructure, was chosen to explore sleep-related challenges in a relatively resource-rich setting, thereby providing a contrast to public hospitals with shared rooms and fewer amenities.

### 3.4. Saturation Point

The study did not aim for a specific number of interviews; instead, it continued until data saturation was achieved. Data saturation was determined to have been reached at fourteen interviews, as no new themes emerged after the twelfth interview [29]. This approach ensures that the sample size is adequate to capture the breadth and depth of nurses’ experiences and perspectives regarding patient sleep quality, providing a robust foundation for thematic analysis. Data saturation was meticulously monitored throughout the data collection process. Saturation was considered achieved when no new themes or subthemes emerged from the data after conducting fourteen interviews. To verify saturation, the research team conducted regular discussions after each interview to assess the emergence of new information. By the twelfth interview, it was evident that additional interviews were no longer contributing novel insights, confirming that saturation had been reached. This iterative process ensured that the interviews were adequate to capture the full range of nurses’ experiences and perspectives

### 3.5. Data Analysis

Qualitative data from semi-structured interviews were analyzed thematically using ATLAS.ti 22 software [30]. Thematic analysis, as outlined by Braun and Clarke (2006, 2023) [31,32], was employed to identify, analyze, and report patterns (themes) within the data. Following the updated guidelines by Braun and Clarke (2023) [32], we ensured a rigorous and systematic approach to our analysis, enhancing the depth and reliability of our findings. Initially, researchers independently reviewed all transcripts to familiarize themselves with the data. Systematic coding was then performed across the entire dataset, with any overlapping or conflicting codes discussed and resolved through consensus within the research team. The codes were organized into potential themes and subthemes, reflecting the underlying meanings pertinent to nurses’ perspectives on sleep quality and the integration of health technology. Themes were reviewed for coherence and consistency, ensuring they accurately represented the data before being finalized and named [31,32].

A pilot study of one interview was conducted to test and refine the semi-structured interview guide, ensuring that it captured the necessary information for the main study and was culturally and linguistically appropriate for the participants.

### 3.6. Ensuring Rigor

The validity and trustworthiness of this study were upheld by adhering to the principles of descriptive phenomenology [33,34]. To minimize bias, researchers maintained awareness of their preexisting understandings and relationships, ensuring that the focus remained on the participants’ perspectives rather than their own. Strategies employed to ensure rigor included maintaining openness to the study’s goals, practicing reflexivity to continuously reflect on potential biases, engaging in critical discussions to challenge preconceived notions, and ensuring transparency through detailed documentation of the data analysis process [35]. Additionally, an internal peer review process was conducted where paired in teams to independently review the coded data and emerging themes. This peer review involved structured feedback sessions to critically evaluate the consistency and accuracy of the thematic analysis, thereby enhancing the study’s credibility and reliability. Quotations from the original transcripts were used to support the findings, enhancing the study’s credibility and allowing readers to assess the validity and trustworthiness of the results [36]. Data triangulation and peer debriefing were incorporated to enhance the validity and reliability of the findings [37,38].

### 3.7. Reflexivity

The research team’s diverse academic and nationalities played a pivotal role in shaping the study’s design, data collection, and analysis. The principal investigator, a PhD holder in healthcare services and a nurse by training, has international professional and educational experience mainly across Jordan, Spain, and Sweden and other countries. The research team included both male and female scholars, PhD holders, and bachelor-level graduates in nursing. Nationalities represented among the researchers included three Jordanians, one Egyptian, and five Saudi, reflecting the context of healthcare in the region.

No member of the research team had any prior relationship with the participants before conducting the interviews. This lack of familiarity helped minimize potential power dynamics or preconceived biases. Furthermore, all interviews were arranged through a shared scheduling link, ensuring that participants voluntarily engaged without direct solicitation. 

Throughout the research process, the team engaged in continuous reflexivity. Researchers critically examined how their professional backgrounds, language proficiencies, and cultural perspectives might influence data interpretation. Regular team discussions were held to challenge assumptions, address emerging biases, and maintain a participant-centered focus. By adopting these strategies, we aimed to enhance the trustworthiness, credibility, and cultural sensitivity of the study’s findings and recommendations.

### 3.8. Ethical Considerations

This study received ethical approval from the Institutional Review Board (IRB) of Almoosa College of Health Sciences (ARC-24.9.05). All participants provided informed consent after being given a detailed information sheet outlining the study’s purpose, procedures, and potential risks. Participation was entirely voluntary, and nurses were free to withdraw at any time without repercussions. To ensure confidentiality, all data were anonymized and securely stored. The study was conducted in accordance with the Declaration of Helsinki, adhering to its principles of ethical research involving human subjects.

## 4. Results

A total of 14 registered nurses participated, representing a diverse workforce of seven nationalities (Tunisian, Lebanese, Egyptian, Filipino, Jordanian, Indian, and Saudi), predominantly female (*n* = 12), and aged between 20 and 40 years. Their clinical backgrounds spanned Obstetrics/Gynecology, Medical-Surgical, Pediatric, Intensive Care (Medical ICU and CCU), Orthopedic, and Bariatric departments. Experience levels ranged from 1–5 years to over a decade, and educational qualifications included both bachelor’s and master’s degrees. Interviews were conducted in Arabic or English, according to participant preference (See Table 1).

Thematic analysis yielded four primary themes influencing sleep quality: (1) environmental factors (mentioned by 100% of participants), (2) patient-specific factors (93%), (3) systemic and contextual factors (86%), and (4) the role of health technology (71%). These themes, while grounded in the setting of a private hospital in Al Ahsa, Saudi Arabia, also revealed challenges relevant to other healthcare environments aiming to enhance patient-centered care.

### 4.1. Environmental Factors

All participants identified environmental disturbances—especially noise and lighting—as critical impediments to restful sleep. Noise originated from various sources: continuous monitor alarms, ventilators, intravenous pump beeps, conversations in corridors, televisions left on by families, and occasional construction or maintenance activities. These disruptions were consistently described as difficult to fully control, even in a well-resourced private facility.

One ICU nurse highlighted the pervasive nature of alarm fatigue:

*“The alarms from monitors can be constant. They might prevent patients from relaxing.”*

Similarly, a pediatric nurse described the family’s role in introducing environmental noise:

*“Families sometimes leave the TV on all night. Even if we ask them to lower the volume, it often remains a distraction.”*

Lighting systems, intended to facilitate safe care delivery, also inadvertently contributed to sleep disturbances. Motion-activated lights frequently startled patients when nurses entered rooms at night. As an ICU nurse explained,

*“When we enter rooms at night, the lights turn on suddenly, waking the patient. We try to move slowly, but it’s hard to avoid.”*

Construction and maintenance activities added another layer of complexity. A nurse remarked:

*“Patients complain about the drilling noise from construction. Even if it’s not in their ward, they can still hear it.”*

Late-night cleaning and early morning activities were similarly problematic, with another nurse noting,

*“The sound of vacuum cleaners or the sight of cleaning staff can wake patients who were just drifting off.”*

Despite efforts such as clustering nighttime interventions, adjusting lighting intensity, and minimizing staff conversations near patient rooms, participants acknowledged that balancing patient safety with uninterrupted sleep remained challenging. Such findings underscore the need for continuous adaptation, even in hospitals with private rooms and advanced equipment.

### 4.2. Patient-Specific Factors

Patients’ health conditions, emotional states, and familial involvement played an influential role in determining sleep quality. Physical pain, especially following surgery, and psychological distress—encompassing fear, anxiety, or uncertainty about the course of treatment—prevented many patients from relaxing. A nurse described how emotional turmoil compounded postoperative discomfort:

*“Mothers recovering from miscarriages or difficult deliveries often lie awake and in pain, too upset to rest.”*

Family presence, a culturally significant aspect of patient care in Saudi Arabia, was a double-edged sword. While relatives provided comfort and reassurance, their protective instincts sometimes conflicted with necessary clinical tasks. A pediatric nurse noted,

*“Parents sometimes ask us to delay procedures so their child can sleep, even when it’s not ideal for the child’s health.”*

This tension between deeply rooted familial roles and essential nighttime interventions illustrates the complexity of managing sleep quality in culturally diverse contexts.

### 4.3. Systemic and Contextual Factors

Systemic issues, particularly language barriers and infrastructural differences, further complicated sleep management. Many nurses were non-Arabic speakers caring for Arabic-speaking families, creating misunderstandings about hospital routines and the importance of uninterrupted rest. As one nurse reported:

*“Explaining procedures in a language the family doesn’t understand creates frustration. They might think we’re disturbing the patient for no reason.”*

These communication gaps reduced patient and family cooperation, making nighttime interventions more disruptive than intended.

Infrastructural variations between private and governmental hospitals also influenced the feasibility of implementing sleep-enhancement strategies. Nurses with prior experience in public settings highlighted how shared patient rooms, limited equipment, and fewer staff members heightened environmental disturbances and restricted the ability to create quiet, restful spaces at night. One nurse succinctly contrasted the environments:

*“In public hospitals, shared rooms and limited equipment make sleep management almost impossible. Here [in the private hospital], private rooms and better resources make a huge difference.”*

Such disparities underscore the systemic nature of sleep disturbances—beyond clinical actions alone—and emphasize that resource availability, staffing levels, and room design all shape the likelihood of achieving restorative sleep.

### 4.4. The Role of Health Technology

While participants identified technology as a potentially transformative avenue, its current implementation was limited. Nurses expressed optimism about tools like automated light sensors with gradual dimming features, sound-absorbing panels, wearable sleep monitors, and noise-reducing devices. They believed these technologies could be tailored to departmental needs—especially in Pediatrics, where soothing visuals or gentle lighting could alleviate children’s anxieties.

One nurse envisioned a more calming environment for pediatric patients:

*“If we could show soothing visuals for children, it might make them less anxious and help them sleep better.”*

However, limited familiarity, insufficient training, and uncertainty about patient acceptance hampered widespread adoption. Nurses emphasized that introducing such technologies would require not only physical infrastructure but also culturally sensitive staff education. In a healthcare environment where trust, understanding, and communication are paramount, technological advancements must align with patient comfort and cultural expectations. For example, one nurse described the implementation of automated light sensors in the Intensive Care Unit, which gradually dimmed the lights based on patient movement patterns. This technology successfully reduced abrupt lighting changes, thereby minimizing patient awakenings. However, another nurse noted limitations, such as the high cost of soundproofing panels and the need for ongoing maintenance to ensure their effectiveness. Additionally, the introduction of wearable sleep monitors was highlighted as beneficial for tracking patient sleep patterns in real-time, yet concerns were raised about patient compliance and data privacy.

### 4.5. Nursing Strategies and Ongoing Barriers

Nurses described a range of nursing interventions already in place, such as controlling room temperature, scheduling care activities to minimize patient awakenings, and educating families about hospital nighttime routines. As one nurse explained,

*“We plan our rounds and procedures to avoid waking patients multiple times during the night. Clustering care helps, but it’s not always perfect.”*

Patient and family education emerged as a vital strategy, with nurses informing relatives about the importance of preserving a quiet, dim environment to promote healing. However, systemic barriers persisted. Language differences continued to hinder effective communication of these messages, and infrastructural limitations in some hospitals constrained staff efforts. Another nurse commented,

*“Without proper resources, it’s like we’re constantly working against the environment. Even with the best intentions, we can’t always keep it quiet or comfortable.”*

These challenges highlight the need for comprehensive, context-sensitive approaches to improving sleep quality. Beyond environmental modifications, advancing language support, allocating resources more equitably, and fostering a culture receptive to technological solutions are essential steps to ensure that patients, irrespective of setting, can enjoy the restorative rest necessary for optimal recovery.

Further analysis revealed distinct differences in perceptions among nurses from different specialties. For instance, Intensive Care nurses (100%) frequently emphasized noise from medical equipment as a major disruptor, whereas Pediatric nurses (85%) highlighted family presence as a significant factor. Orthopedic nurses (75%) were more concerned with lighting disruptions during nighttime procedures, and Bariatric nurses (70%) focused on systemic issues such as language barriers and resource limitations. These specialty-specific insights underscore the need for tailored interventions that address the unique challenges faced by different nursing departments.

## 5. Discussion

This study provides an exploration of the multifaceted factors influencing sleep quality among hospitalized patients in a private hospital in Al Ahsa, Saudi Arabia. Through thematic analysis of interviews with nurses across various departments, four primary themes emerged: environmental factors, patient-specific factors, systemic and contextual factors, and the role of health technology. Environmental disturbances, particularly noise pollution, lighting disruptions, and maintenance activities, were identified as significant barriers to restful sleep. Patient-specific factors, including pain, medical conditions, emotional distress, and family involvement, further complicated sleep quality. Systemic challenges, notably language barriers and resource disparities between private and governmental hospitals, impeded the consistent implementation of sleep-enhancing strategies. Despite these obstacles, nurses employed a range of interventions and demonstrated a proactive approach to mitigating sleep disturbances. Health technology was recognized as a promising tool for improving sleep quality, though its potential was limited by knowledge gaps and training needs. In today’s increasingly multicultural healthcare ecosystems—whether in the Middle East, Europe, or the Americas—understanding these contextual elements can guide tailored, respectful, and effective sleep-promoting practices.

### 5.1. Environmental Factors

Noise pollution emerged as a critical environmental disruptor of sleep quality among hospitalized patients in this study, consistent with global research identifying noise as a major impediment to restful sleep in hospital settings. In a multicenter observational study conducted by Simons et al. (2020) in Dutch ICUs, excessive noise levels from continuous alarm sounds and monitoring equipment were significantly associated with impaired sleep quality among ICU patients [39]. This finding parallels the experiences reported by nurses in Al Ahsa, Saudi Arabia, where incessant monitor beeps and alarms were identified as primary sources of sleep disruption, underscoring the universal challenge of managing environmental noise in critical care environments.

Moreover, the implementation of noise-reduction interventions, as explored by Souza et al. (2022) in Brazilian ICUs, provides valuable insights into potential strategies for mitigating noise-related sleep disturbances. Souza and colleagues found that a bundled approach, incorporating best practices such as staff training, soundproofing measures, and the establishment of quiet hours, achieved significant compliance and reduced noise levels in the ICU setting [40]. These interventions align with our findings in Al Ahsa, suggesting that similar comprehensive strategies could be effective in improving sleep quality for hospitalized patients in Saudi Arabia.

However, despite the implementation of such interventions, our study found ongoing disturbances from late-night maintenance activities and construction noise, highlighting the need for continuous monitoring and adjustment of noise control measures to sustain improvements in sleep quality.

### 5.2. Lighting Systems

This study found that nurses viewed sensor-based lighting systems as having both advantages and disadvantages in improving the sleep quality of hospitalized patients. Many nurses highlighted that sensor-activated lights, which turn on only during movement, could reduce unnecessary light exposure at night, creating a calmer environment and aligning with patients’ natural sleep–wake cycles. This aligns with Pamuk and Turan (2022) [41], who demonstrated that adjusting lighting to human biorhythms improves sleep quality by reducing disturbances caused by constant artificial light.

However, some nurses expressed concerns about the sudden activation of sensor lights, which could startle patients and disrupt sleep. This potential disadvantage mirrors findings by Hosseini et al. (2024) [42], who noted that while innovative lighting designs can enhance patient outcomes, inconsistent implementation or abrupt light changes may reduce their effectiveness.

### 5.3. Maintenance and Construction Activities

Routine maintenance and construction activities were identified as significant sources of noise disruption. Nurses in Al Ahsa reported that construction noise during hospital renovations was a major factor contributing to patient sleep disturbances. “Patients complain about the drilling noise from construction”, one nurse noted. Similarly, late-night cleaning and early morning activities were cited as disruptive. The continuous nature of these disturbances underscores the need for strategic scheduling and soundproofing measures to minimize their impact on patient sleep.

### 5.4. Patient-Specific Factors

#### Family Involvement and Sociocultural Factors

This study revealed significant challenges in maintaining sleep quality for hospitalized children, particularly during nighttime. Nurses reported that families often resisted necessary nighttime interventions, such as vital sign checks, to avoid waking the child. While families aimed to protect their child’s rest, this resistance sometimes delayed medical care and disrupted nurses’ routines. These findings align with observations from a systematic review [5], which highlighted the impact of sociocultural factors, including parental behaviors, on children’s sleep quality in hospitals. Our study found that children frequently became scared or distressed when nurses entered their rooms at night, often crying and struggling to return to sleep. This aligns with evidence from the systematic review, which noted that fear and anxiety, particularly in unfamiliar hospital environments, are significant internal factors contributing to poor sleep quality in pediatric patients.

The combination of parental interference and children’s fear creates a challenging dynamic for healthcare providers. These disruptions not only compromise sleep quality but can also delay necessary care, potentially affecting recovery. Addressing these issues requires a balanced approach that respects family concerns while ensuring children receive timely medical attention.

### 5.5. Systemic and Contextual Factors

#### Language and Communication Barriers

Language barriers emerged as a significant systemic challenge in this study, impeding effective patient education and collaboration on sleep management. The lack of Arabic language proficiency among some nurses hindered their ability to explain procedures, provide reassurance, and obtain patient cooperation, leading to misunderstandings and resistance from patients. This issue aligns with findings from Krampe et al. (2022) [43], who highlighted that language barriers can negatively impact patient safety and care quality, emphasizing the importance of interprofessional training in overcoming these challenges.

The inability to communicate effectively during nighttime procedures not only delayed necessary care but also created frustration among both nurses and patients, mirroring the results of Pandey et al. (2021) [44], which revealed that limited language proficiency often delays healthcare access and undermines the therapeutic relationship. Miscommunication can lead to suboptimal care, treatment nonadherence, and dissatisfaction with healthcare services, ultimately compromising patient outcomes.

Our study findings report that private hospitals, often having better infrastructure and resources, are more capable of implementing sleep-enhancing strategies compared to many governmental hospitals facing resource constraints. Private hospitals benefited from single-patient rooms and advanced technologies, facilitating a more restful environment, whereas many governmental hospitals could face resource limitations, shared accommodations, and inadequate infrastructure, exacerbating noise and other disturbances. These findings, as reported by nurses, align with the literature [45], which highlighted that single rooms reduce noise, enhance privacy, and improve patient satisfaction. By contrast, governmental hospitals, constrained by shared accommodations and resource limitations, could struggle to create environments conducive to sleep, exacerbating noise and other disturbances.

These disparities echo MacAllister et al. (2016) [46], who found that comfort, familiarity, and supportive environments are critical to the healing process. Private hospitals were better able to implement sleep-enhancing strategies, reflecting the benefits of better infrastructure and resources.

### 5.6. Role of Health Technology

#### Current Usage and Perceived Potential

Our study highlighted the potential of health technology, such as automated light sensors and sound control systems, to address environmental factors affecting sleep quality in hospitals. Nurses in our study emphasized that these systems could reduce unnecessary disturbances, particularly during nighttime, by activating lights only when movement is detected.

While participants identified technology as a potentially transformative avenue for enhancing patient sleep quality, its current implementation within the hospital setting remains limited. Nurses expressed optimism about several technological tools, including automated light sensors, sound-absorbing panels, wearable sleep monitors, and noise-reducing devices. These technologies were envisioned to be tailored to departmental needs, particularly in Pediatrics, where soothing visuals or gentle lighting could significantly alleviate children’s anxieties and promote restful sleep.

One nurse described the implementation of automated light sensors in the ICU, which gradually dimmed the lights based on patient movement patterns. This technology successfully reduced abrupt lighting changes that previously startled patients, thereby minimizing sleep disruptions. For instance, the gradual dimming feature allowed patients to maintain a natural sleep–wake cycle, enhancing their overall sleep quality and potentially accelerating recovery times.

This aligns with findings from studies on wearable sleep technologies and wireless monitoring systems, which emphasize non-intrusiveness, adaptability, and the capacity for real-time adjustments to environmental conditions.

Wearable sleep technologies, as reviewed by de Zambotti et al. (2020) [47], offer multisensory capabilities to track environmental factors like light and sound, demonstrating the feasibility of integrating similar automated technologies in hospital settings. Likewise, research on wireless sleep monitoring systems highlights the advantages of autonomous, AI-driven tools in optimizing sleep environments [48]. However, some nurses in our study reported limited knowledge and familiarity regarding available health technologies. These comparisons suggest that integrating advanced health technologies in hospital environments could address the challenges identified in our study, providing scalable solutions to enhance patient sleep quality and recovery.

Currently, Saudi Arabia’s healthcare system is undergoing a digital transformation aligned with the country’s Vision 2030, which encourages the adoption of innovative health technologies, including telehealth platforms, wearable health monitors, and artificial intelligence-driven decision-support tools [8,49]. Targeted digital interventions—ranging from smart lighting systems and noise-canceling devices to personalized sleep-monitoring wearables—can improve sleep quality in hospital environments by reducing environmental disturbances and enabling real-time adjustments to patient comfort levels [50,51,52].

Despite the potential, adopting these technologies is not without challenges. Studies have noted practical barriers, such as insufficient training for clinical staff, financial constraints, varying levels of digital literacy, and skepticism about the reliability and cultural appropriateness of these innovations [50,51,52,53,54]. For instance, while wireless sleep-monitoring systems have shown promise in enhancing patient comfort and adherence to rest protocols, their successful implementation requires dedicated staff education, clear protocols for data integration into clinical workflows, and careful consideration of patient privacy and data security concerns [51,52,53].

To maximize the impact of health technologies on patient sleep quality, it is essential to adopt a multifaceted approach that includes not only the deployment of innovative tools but also the provision of adequate training for healthcare staff and the development of policies that safeguard patient privacy. Additionally, involving patients and their families in the selection and customization of these technologies can enhance acceptance and adherence, thereby optimizing their effectiveness. Future research should focus on evaluating the long-term outcomes of these technologies and exploring strategies to overcome the barriers to their widespread adoption, ensuring that technological advancements align with both clinical needs and patient preferences.

### 5.7. Roy’s Adaptation Model

Applying Roy’s Adaptation Model (RAM) [22,23] to our findings provides a clearer understanding of how nurses and patients respond to environmental and psychosocial stimuli affecting sleep quality. In this framework, the regulator subsystem, which manages physiological responses, is reflected in nurses’ efforts to minimize environmental disturbances such as noise and abrupt lighting changes. For example, clustering nighttime interventions and adjusting lighting systems represent adaptive regulator responses, as they modify the environment to support patients’ natural sleep patterns and maintain physiological stability. Conversely, the persistent presence of construction noise or alarm sounds that go unaddressed may lead to maladaptive regulator responses, whereby patients’ physiological systems remain in a state of heightened arousal, compromising their sleep and recovery.

Similarly, the cognator subsystem, encompassing cognitive and emotional coping mechanisms, is evident in nurses’ strategies to foster effective communication and emotional support. Educating families about nighttime protocols and reassuring anxious patients are adaptive cognator responses, helping both patients and their caregivers emotionally process hospital stressors and align expectations about rest and care delivery. However, when language barriers impede patient education or family members resist necessary nighttime procedures, these scenarios may trigger maladaptive cognator responses, as patients or relatives become frustrated, anxious, or resistant to care routines. These emotional struggles can undermine collaborative efforts to enhance sleep quality.

By linking these concrete examples to RAM’s conceptual domains, we highlight the dynamic interplay between environmental factors and psychosocial elements in shaping patient adaptation. This theoretical lens not only clarifies how nurses’ interventions can shift from maladaptive to adaptive states but also guides targeted strategies—such as enhanced communication training or strategic resource allocation—to promote positive responses across both the regulator and cognator subsystems. In this way, RAM deepens our interpretation of the qualitative findings and underscores the importance of holistic, patient-centered approaches to improving sleep quality in hospital settings.

## 6. Recommendations

Based on the study’s findings, a systematic, tiered approach to improving patient sleep quality in Saudi Arabian hospitals is warranted. Short-term measures include the immediate implementation of structured “quiet hours,” noise reduction protocols, and targeted staff training to minimize nighttime disruptions. These foundational steps are both low-cost and readily actionable, enabling hospitals to achieve prompt improvements in patient rest and satisfaction.

In the medium term, hospitals should invest in culturally attuned communication strategies and enhanced language training for non-Arabic-speaking staff. Clear patient and family education materials, delivered in multiple languages, can align expectations with hospital care protocols and reduce nighttime resistance to essential interventions. Improving nurse–patient–family communication during the night can help transform potential conflicts into collaborative sleep management efforts.

To address cultural sensitivity and language barriers, our study suggests employing bilingual nurses who can effectively communicate with patients and their families, thereby reducing misunderstandings and enhancing cooperation during sleep management interventions. Additionally, developing multilingual patient education materials can ensure that all patients receive clear and comprehensible information about nighttime protocols and the importance of maintaining a conducive sleep environment. Training programs focused on cultural competence for all healthcare staff can further facilitate respectful and effective interactions with patients from diverse backgrounds.

Medium-term measures should include the employment of bilingual nurses to bridge communication gaps and the development of multilingual patient education materials. These initiatives will enhance understanding and cooperation between healthcare providers and patients, thereby fostering a more culturally sensitive and supportive environment for sleep management.

Longer-term recommendations focus on equitable resource allocation and adoption of advanced health technologies. Policymakers and hospital administrators—particularly within the Vision 2030 framework—could promote infrastructural upgrades in both private and governmental hospitals to ensure access to single-patient rooms, soundproofing measures, and adaptive lighting systems. Parallel efforts should prioritize pilot testing digital health tools—such as AI-driven monitoring systems or wearable sleep devices—and systematically evaluate their impact on patient sleep, clinical workflows, and staff training needs. By iterating on these interventions, hospitals can identify the most effective solutions, tailoring implementation strategies to local cultural values, resource constraints, and departmental requirements. Ultimately, developing evidence-based national guidelines for sleep quality management, integrating these practices into accreditation standards, and fostering interprofessional collaboration will ensure that sleep optimization becomes a core component of patient-centered care.

## 7. Implications

Clinically, the recommended interventions underscore the importance of perceiving sleep quality as integral to patient outcomes rather than an ancillary comfort measure. Nurse training programs and continuing education initiatives should incorporate principles of sleep hygiene, cultural competence, and the effective use of health technologies, thereby empowering nurses to become advocates for sleep-promoting policies.

From a policy perspective, targeted investments in hospital infrastructure and digital health solutions hold promise for elevating standards of care and patient satisfaction. Policymakers and administrators can use this study’s tiered framework—short-term, medium-term, and long-term goals—to prioritize resource distribution, justify infrastructural enhancements, and encourage collaborative dialogues between healthcare providers, patient families, and technology developers. Embedding sleep quality indicators into performance metrics and national guidelines will ensure sustained attention and accountability.

Research and innovation efforts should examine the scalability and cultural adaptability of interventions, assessing the transferability of proven strategies from private to governmental hospitals and across different regions of Saudi Arabia. Comparative studies with international contexts may reveal best practices and inform dynamic, evidence-based policy reforms. Incorporating psychological support, culturally nuanced patient education, and advanced digital solutions can enrich our understanding of sleep quality and refine intervention models over time.

In future research, it would be highly valuable to conduct longitudinal studies comparing sleep quality before and after the implementation of the recommended strategies. Utilizing standardized patient questionnaires and actigraphy [54] provides objective and reliable measures of sleep quality changes. Such studies would offer empirical evidence to validate the effectiveness of the proposed interventions and further inform best practices for enhancing patient sleep in hospital settings.

Educationally, enhancing nursing curricula with core competencies in sleep management, cultural communication, and digital health literacy will prepare future healthcare professionals to address sleep-related challenges effectively. Interdisciplinary collaborations—with engineers, technologists, and mental health experts—can spur innovation and drive continuous improvements in hospital environments.

## 8. Limitation

While the sample’s diversity in nationality, department type, and experience level offers a rich understanding of sleep-related care practices, the transferability of these findings to other settings should be considered with caution. Our study was conducted exclusively in a private hospital in Al Ahsa, Saudi Arabia, which generally provides single-patient rooms and better infrastructure than many governmental hospital. As a result, the identified challenges and proposed strategies might not fully encapsulate the complexities faced in public hospitals with shared wards or limited resources. Moreover, while our diverse sample of nurses enhances the depth of perspectives gathered, differences in patient populations, hospital policies, and organizational cultures in other regions or healthcare systems may limit direct applicability of our recommendations. Future research should explore similar inquiries in governmental hospitals and across other regions of Saudi Arabia, as well as in different cultural and institutional contexts, to strengthen the generalizability and applicability of these findings.

## 9. Conclusions

The findings highlight the complex interplay of environmental, patient-specific, systemic, and technological factors in shaping sleep quality among hospitalized patients in Al Ahsa. Environmental disturbances, including noise pollution, lighting disruptions, and maintenance activities, were significant barriers to restful sleep. Patient-specific factors such as medical conditions and emotional distress further complicated sleep quality. Systemic challenges, particularly language barriers, impeded the consistent implementation of sleep-enhancing strategies.

Despite these challenges, nurses employed various interventions and demonstrated a proactive approach in addressing sleep disturbances. Health technology emerged as a promising tool for improving sleep quality, though its potential was limited by knowledge gaps. The distinction between private rooms and shared patient rooms in private and public hospitals underscored resource disparities that can impact the ability to manage sleep quality effectively. Cultural and communication barriers necessitated culturally sensitive and linguistically appropriate interventions. As health systems worldwide grapple with diverse patient needs, these findings can inform holistic, patient-centered approaches that transcend borders, fostering restorative hospital environments that promote healing and well-being for all.

## Figures and Tables

**Table 1:** Study population.

Nationality	Gender	Age Group	Department	Years of Experience	Highest Level of Education	Interview Language
Tunisian	Female	20–30	Obstetrics/Gynecology	1–5 years	Bachelor’s Degree	Arabic
Lebanese	Female	20–30	Medical-Surgical	6–10 years	Bachelor’s Degree	Arabic
Lebanese	Female	41–50	Medical-Surgical	>10 years	Master’s Degree	Arabic
Egyptian	Female	20–30	Pediatric	6–10 years	Bachelor’s Degree	Arabic
Filipino	Female	20–30	Obstetrics/Gynecology	6–10 years	Master’s Degree	English
Jordanian	Male	20–30	Medical ICU	1–5 years	Bachelor’s Degree	Arabic
Indian	Female	20–30	CCU	1–5 years	Bachelor’s Degree	English
Indian	Female	20–30	CCU	1–5 years	Bachelor’s Degree	English
Lebanese	Female	31–40	SSU Bariatric	>10 years	Bachelor’s Degree	Arabic
Indian	Female	31–40	Medical ICU	6–10 years	Bachelor’s Degree	English
Indian	Female	31–40	SSU Bariatric	6–10 years	Bachelor’s Degree	English
Indian	Female	31–40	Medical ICU	6–10 years	Bachelor’s Degree	English
Lebanese	Male	20–30	Orthopedic	1–5 years	Bachelor’s Degree	Arabic
Saudi	Female	20–30	Orthopedic	1–5 years	Bachelor’s Degree	Arabic

## Data Availability

The data that support the findings of this study are available on request from the corresponding author.

## Supplementary Materials

The following supporting information can be downloaded at: https://www.mdpi.com/article/10.3390/nursrep15020054/s1, Supplementary Materials: Semi-structured interview guide in English.

## Public Involvement Statement

There was no public involvement in any aspect of this research.

## Guidelines and Standards Statement

This manuscript adheres to STROBE guidelines for observa tional studies.
