# Supplementary material for: Nurses’ Perspectives on the Sleep Quality of Hospitalized Patients in Al Ahsa, Saudi Arabia
Source: Nurs Rep. 2025 Feb 4;15(2):54. doi: 10.3390/nursrep15020054 (PMC11858348; doi:10.3390/nursrep15020054)
Supplement: Supplementary file 1 [file nursrep-15-00054-s001.zip › nursrep-3398659-supplementary.pdf]

Supplementary Materials: Semi-Structured Interview Guide

Introduction:

- Thank the participant for their time and explain the purpose of the interview.
- Provide a brief overview of the study, emphasizing the confidentiality of the responses and the voluntary nature of participation.
- Obtain verbal consent to proceed with the interview and to record the session.

Warm-Up Questions:

1. Can you tell me about your current role and responsibilities in the hospital?
2. How long have you been working as a nurse, and how much of that time has been spent in inpatient care?
3. How often do you work night shifts, and what does your typical night shift routine involve?

Main Questions:

1. **Understanding Sleep Quality:**
    - From your experience, what factors do you believe most significantly impact the sleep quality of hospitalized patients during night shifts?
    - How do you assess whether a patient is having trouble sleeping? Are there specific signs or behaviors you look for?
2. **Challenges in Managing Sleep Quality:**
    - What challenges do you encounter when trying to help patients achieve better sleep during their stay?
    - How do hospital routines, such as nighttime medical procedures or noise levels, affect patients' sleep?
3. **Current Strategies and Interventions:**
    - What strategies or interventions have you used or observed to improve sleep quality among patients? How effective do you think these have been?
    - Are there any non-pharmacological interventions (e.g., earplugs, eye masks, adjusting room lighting) that you regularly use or recommend?
4. **Role of Health Technology:**
    - In your opinion, how can health technology be utilized to improve sleep quality for patients?

    - Are you aware of or have you used any technological tools (e.g., sleep monitoring systems, environmental control systems) to assist in managing sleep disturbances? If so, what has been your experience with them?

5. **Attitudes Toward Technology:**

    - How do you feel about the integration of new technologies in patient care, particularly in relation to improving sleep quality?
    - What potential benefits and challenges do you foresee in adopting these technologies on a broader scale within the hospital?

6. **Suggestions and Recommendations:**

    - Based on your experience, what changes or improvements would you suggest to enhance sleep quality in hospitalized patients?
    - How do you think these changes could be implemented in your current work environment?

Closing:

- Is there anything else you would like to add about sleep quality in hospitalized patients or the use of health technology in this area?
- Thank the participant again for their time and valuable insights. Remind them of the confidentiality of their responses and the next steps in the study.

Notes for the Interviewer:

- Use follow-up questions or prompts as needed to encourage the participant to elaborate on their responses.
- Maintain a neutral tone and avoid leading questions to ensure unbiased data collection.
- Ensure that the interview remains focused on the study objectives, but allow flexibility for the participant to share relevant experiences.
